# Integrated Solid-Phase Extraction, Ultra-High-Performance Liquid Chromatography–Quadrupole-Orbitrap High-Resolution Mass Spectrometry, and Multidimensional Data-Mining Techniques to Unravel the Metabolic Network of Dehydrocostus Lactone in Rats

**DOI:** 10.3390/molecules27227688

**Published:** 2022-11-09

**Authors:** Yingying Tian, Beibei Ma, Chuang Liu, Xinyue Zhao, Shangyue Yu, Yilin Li, Shiqiu Tian, Hailuan Pei, Zijian Wang, Zeping Zuo, Zhibin Wang

**Affiliations:** 1 School of Chinese Materia Medica, Beijing University of Chinese Medicine, Beijing 100029, China; 2 Research Institute of Beijing Tongrentang Co., Ltd., Beijing 100079, China

**Keywords:** dehydrocostus lactone (DL), UHPLC–Q-Orbitrap HRMS, solid-phase extraction (SPE), data-mining techniques, metabolic network

## Abstract

Dehydrocostus lactone (DL) is among the representative ingredients of traditional Chinese medicine (TCM), with excellent anticancer, antibacterial, and anti-inflammatory activities. In this study, an advanced strategy based on ultra-high-performance liquid chromatography–quadrupole-Orbitrap high-resolution mass spectrometry (UHPLC–Q-Orbitrap HRMS) was integrated to comprehensively explore the metabolic fate of DL in rats. First, prior to data collection, all biological samples (plasma, urine, and feces) were concentrated and purified using solid-phase extraction (SPE) pre-treatment technology. Then, during data collection, in the full-scan (FS) data-dependent acquisition mode, FS-ddMS^2^ was intelligently combined with FS-parent ion list (PIL)-dynamic exclusion (DE) means for targeted monitoring and deeper capture of more low-abundance ions of interest. After data acquisition, data-mining techniques such as high-resolution extracted ion chromatograms (HREICs), multiple mass defect filters (MMDFs), diagnostic product ions (DPIs), and neutral loss fragments (NLFs) were incorporated to extensively screen and profile all the metabolites in multiple dimensions. As a result, a total of 71 metabolites of DL (parent drug included) were positively or tentatively identified. The results suggested that DL in vivo mainly underwent hydration, hydroxylation, dihydrodiolation, sulfonation, methylation, dehydrogenation, dehydration, N-acetylcysteine conjugation, cysteine conjugation, glutathione conjugation, glycine conjugation, taurine conjugation, etc. With these inferences, we successfully mapped the “stepwise radiation” metabolic network of DL in rats, where several drug metabolism clusters (DMCs) were discovered. In conclusion, not only did we provide a refined strategy for inhibiting matrix effects and fully screening major-to-trace metabolites, but also give substantial data reference for mechanism investigation, in vivo distribution visualization, and safety evaluation of DL.

## 1. Introduction

**Figure 1:**
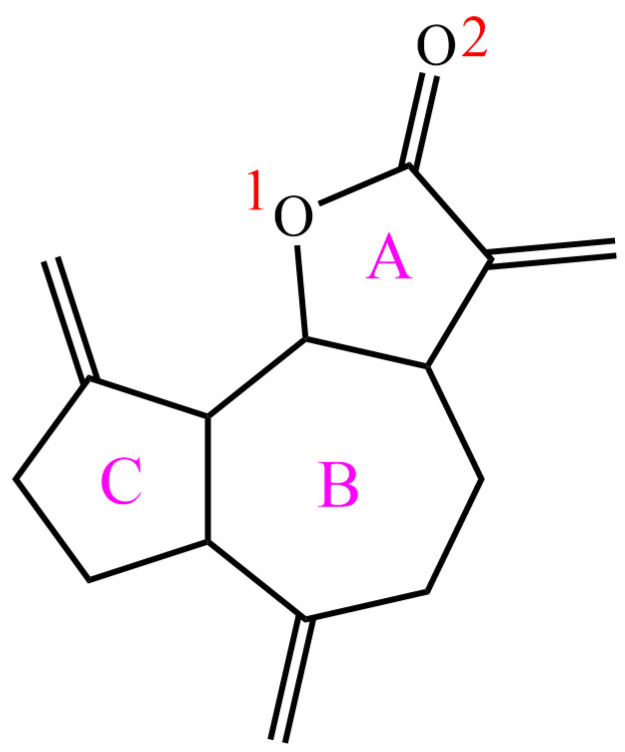
Chemical structure of DL.

Dehydrocostus lactone (DL), extracted and isolated from the roots of *Aucklandia lappa* Decne., *Vladimiria souliei* (Franch.) Ling, *Saussurea costus* (Falc.) Lipsch. and other plants of the Asteraceae family [1,2,3], is a natural sesquiterpene lactone (Figure 1). In recent years, DL has been proven to exhibit broad and outstanding biological activities (such as anticancer, anti-inflammatory, antitumor, and antibacterial properties) [4,5,6,7], which makes it a potential candidate against lung cancer, laryngocarcinoma, gastrinoma, ulcerative colitis, airway allergic inflammation, and acute lung injury [8,9,10,11,12,13]. Reportedly, DL could not only directly target IKKβ with its own crossing blood–brain barrier function and thereby inactivate the NF-κB/COX-2 signaling pathway to antagonize glioma [14], but also inhibit the proliferation of human chronic myeloid leukemia cells through pinpointing Bcr/Abl-JAK/STAT signaling channel [15]. Therefore, DL has been consistently featured as a focal point of interest in both medical research and clinical applications. However, the biotransformation process of DL in vivo has been rarely disclosed, which would hinder its further mechanism exploration and exploitation for use. So, a pure, rapid, and efficient analytical workup is urgently required to thoroughly elucidate its metabolic fate in vivo.

Any form of transformation and presence of the drug after absorption into a body may be an essential component towards its efficacy. Hence, recognizing all the metabolites produced by a drug in vivo and defining their structures is an indispensable step for drug development. Ultra-high-performance liquid chromatography–high-resolution mass spectrometry (UHPLC–HRMS), a powerful analytical technique that has been developed over the past years, is widely used for the metabolic analysis of various Chinese herbal compounds and monomers with its superiority of high sensitivity, high selectivity, and high mass accuracy [16,17,18]. Yet, the signals of numerous trace metabolites are highly susceptible to being overwhelmed by the matrix effects and background noise generated during sample detection. In addition, although many post-acquisition data processing methods have been manifested [19,20,21], such as isotope pattern filtering (IPF), background subtraction (BS), and mass defect filtering (MDF), there is still a lack of effective multimethod integration in the practical application process. Consequently, an advanced strategy of “purification-high quality acquisition-multidimensional data-mining” was developed to address the shortcomings of the existing “pre-acquisition”, “in-acquisition” and “post-acquisition” processes for LC–MS/MS-based drug metabolism analysis, which was verified and expected to be expanded for the in vivo metabolic profiling of complicated ingredients of traditional Chinese medicine (TCM).

In fact, a refined strategy based on UHPLC–Q-Orbitrap HRMS analysis was established to comprehensively capture and characterize the major-to-trace metabolites of DL in rat plasma, urine, and feces: (1) solid-phase extraction (SPE) methodology was adopted for the fast purification and concentration of pre-acquisition biological samples to minimize matrix effects; (2) in the full-scan (FS) data-dependent acquisition (DDA) mode, FS-ddMS^2^ was rationally coupled with FS-parent ion list (PIL)-dynamic exclusion (DE) for more complete and in-depth targeting analysis; (3) several technologies including diagnostic product ions (DPIs), neutral loss fragments (NLFs), high-resolution extracted ion chromatograms (HREICs), and multiple mass defect filters (MMDFs) were consolidated for data mining in multiple dimensions. With these methodological improvements, the biotransformation forms of DL in rats were successfully probed in detail for the first time and based on which the metabolic network of DL in vivo was mapped.

## 2. Results

### 2.1. The Construction and Interpretation of Analysis Strategy

**Figure 2:**
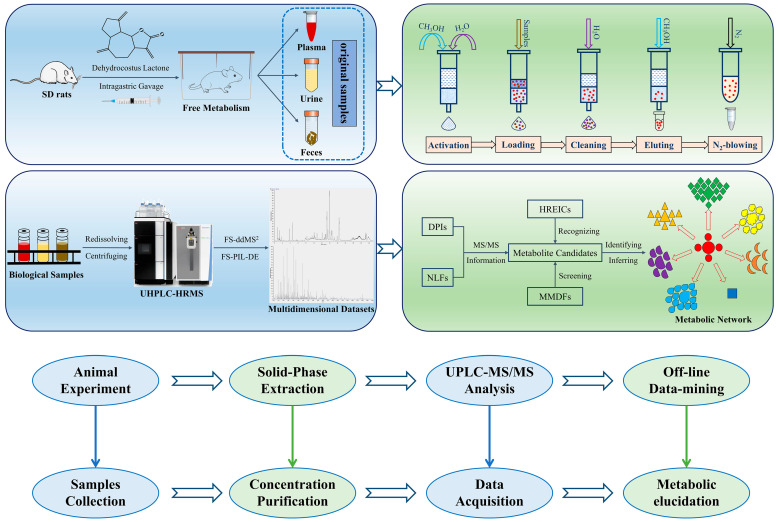
Schematic representation of analytical strategy and workflow.

In this study, the whole analytical workflow was divided into four modules: animal experiment, sample purification, instrumental analysis, and data processing (Figure 2). First, all raw biological samples (i.e., plasma, urine, and feces) from rats orally administered DL were collected. Second, all the samples were quickly enriched and purified using the SPE method in order to remove a majority of impurities and interferents such as salts, proteins, and lipids, so that the matrix effects and background noise could be both reduced. Then, by utilizing the unique DDA mode of Q-Orbitrap HRMS, FS-ddMS^2^ and FS-PIL-DE were innovatively coordinated to enlarge the width and depth of the trapped ions, which could indirectly achieve the target recognition of small-molecule metabolites. Lastly, multiple data-mining techniques such as DPIs, NLFs, HREICs, and MMDFs were synthesized to screen and affirm the candidate metabolites in diverse dimensions, with preliminary identification based on chromatographic retention times, exact mass measurements, and regular fragmentation patterns, from which the metabolic network of DL in rats was further mapped. The elaborate scheme of the developed analytical strategy was presented in Figure 2.

### 2.2. Establishment of the MMDF Screening Method

After obtaining the multidimensional LC–MS/MS datasets of all the biological samples, it is undoubtedly crucial to effectively and accurately determine the metabolite candidates from the enormous and redundant database. Taking into account the literature survey, metabolic pathway speculation, and HREIC verification, the MDF templates were set up as listed in Table 1. The mass error for small molecules generated by mass defects was set to ± 50 mDa around the mass defect of the filter template over a mass range of 50 Da above and below the accurate mass of each individual MDF template. The settings of the upper several filter templates adequately accounted for the involvements of drug filters, substructure filters, and conjugation filters, so that endogenous substances could be maximally eliminated with some low-level or unpredictable metabolites then picked out.

### 2.3. Analysis of the Fragmentation Behaviors of DL in the Positive Ion Mode

**Figure 3:**
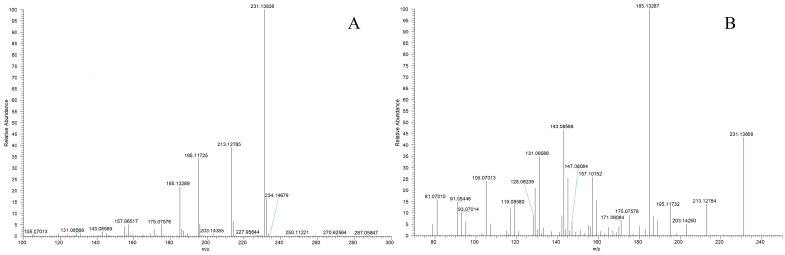
ESI–MS*^n^* spectra of DL in the positive ion mode: (**A**) MS^1^ spectrum; (**B**) MS^2^ spectrum.

**Figure 4:**
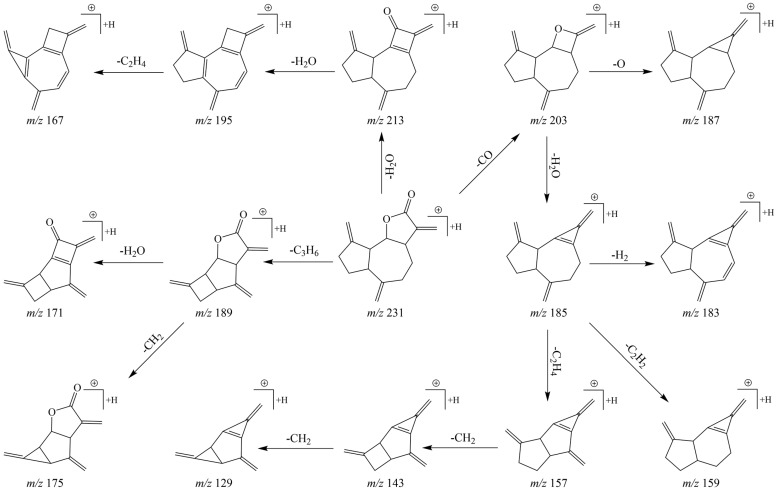
The proposed fragmentation pathways for DL.

In the post-acquisition handling phase of the analytical procedure, DPIs and NLFs were ascertained with primary reference to the characteristic fragmentation behaviors of DL. As illustrated in Figure 3, after UHPLC–MS/MS analysis, DL appeared in the ESI–MS^1^ spectrum in the positive ion mode, with *m*/*z* 231.13838 as molecular ion [M + H]^+^ (C_15_H_19_O_2_, 1.83 ppm). Thereafter, peaks of distinct fragment ions at *m*/*z* 203, *m*/*z* 185, *m*/*z* 157, *m*/*z* 143, and *m*/*z* 129 were recognized and were attributed to successive loss of CO, H_2_O, 2CH_2_, CH_2_, and CH_2_. Further, DPIs at *m*/*z* 213 ([M + H – H_2_O]^+^), *m*/*z* 195 ([M + H – 2H_2_O]^+^), *m*/*z* 167 ([M + H – 2H_2_O – 2CH_2_]^+^), *m*/*z* 189 ([M + H – 3CH_2_]^+^), and *m*/*z* 171 ([M + H – 3CH_2_ – H_2_O]^+^) were also observed as important products in the ESI–MS^2^ spectra. Thus, the constant loss of H_2_O (18 Da) and CH_2_ (14 Da) with regularity was considered as the principal NLFs of DL. The characteristic fragmentation pathways of DL were proposed in Figure 4. Given the similarity between the substructure or parent nucleus of the metabolites and the original drug, the DPIs and NLFs determined so far certainly would serve as significant aids for structure qualification.

### 2.4. Structural Identification of DL Metabolites

After data acquisition by UHPLC–Q–Orbitrap HRMS, the total ion chromatograms (TICs) presented by blood, urine and feces samples of the drug-treated group must be, respectively, compared to the control group in the Xcalibur 4.3 visualized datasets so that interference from endogenous substances or impurities could be excluded for more accurately identifying metabolites.

The metabolites M0, M53, M57, M61, and M69 produced the same [M + H]^+^ as DL at *m*/*z* 231.13796 (C_15_H_19_O_2_, error ≤ ±3.10 ppm), with elution times of 13.34, 9.22, 9.58, 9.70 and 10.38 min, respectively. By comparison with the ESI–MS*^n^* spectra and retention time of the DL reference standard, M0 could be definitively identified as dehydrocostus lactone (DL). Accordingly, due to having analogous DPIs (*m*/*z* 213, 195, 185) and NLFs (18 Da and 14 Da), the rest of the metabolites were positional isomers derived from DL conversion.

Metabolite M26 eluted at 6.38 min yielded [M + H]^+^ at *m*/*z* 249.14856 (C_15_H_21_O_3_, 2.93 ppm), showing 18 Da (one H_2_O molecule) higher than DL. Moreover, the NLFs of 18 Da (*m*/*z* 249→*m*/*z* 231→*m*/*z* 213) along with 28 Da (*m*/*z* 231→*m*/*z* 203) in the ESI–MS^2^ spectrum further indicated that M26 was a hydration product of DL, as well as the related literature could confirm the above inference [22].

Metabolites M27, M31, M37, M45, and M52 were separated by elution at 6.44, 6.54, 7.19, 7.82, and 8.77 min, respectively, and had an identical [M + H]^+^ at *m*/*z* 410.16322 (C_20_H_28_O_6_NS, error ≤ ±3.20 ppm), which was 161 Da (C_5_H_7_NO_3_S) higher than that of M26. Thus, they might be the isomeric products of M26 after N-acetylcysteine conjugation. The [NAC − SH]^+^ (C_5_H_8_O_3_N) at *m*/*z* 130 and the [M – NAC – H_2_O]^+^ at *m*/*z* 229, which were common to them in the ESI–MS^2^ spectrum, also provided sufficient evidence for our assumption. Both M28 and M32 gave rise to [M + H]^+^ at *m*/*z* 428.17379 (C_20_H_30_O_7_NS, error ≤ ±2.60 ppm), with 18 Da higher than M27. The characteristic DPI [M – NAC – H_2_O]^+^ at *m*/*z* 247 suggested that M28 and M32 were hydration products of M27.

Metabolites M1, M2, and M12, which were eluted at 3.63, 3.75, and 5.63 min, generated the same [M + H]^+^ at *m*/*z* 368.15262 (C_18_H_26_O_5_NS, error ≤ ±5.00 ppm). Their molecular weight was 119 Da (C_3_H_5_O_2_NS) higher than that of M26, exhibiting a DPI at *m*/*z* 229 with NLFs of 18 Da and 28 Da (*m*/*z* 368→*m*/*z* 350→*m*/*z* 322), thus these three metabolites could be initially defined as cysteine conjugation products of M26. Similarly, M9 (*m*/*z* 386.16319, C_18_H_28_O_6_NS), which resulted in a DPI of *m*/*z* 368, was attributed to the further hydration product of M1.

M18, eluted at 5.92 min, presented a [M + H]^+^ ion at *m*/*z* 554.21676 (C_25_H_36_O_9_N_3_S, 2.21 ppm), which was 305 Da (C_10_H_15_O_6_N_3_S) larger than M26. According to its ESI–MS^2^ spectrum, the presence of the fragment ion [M – GSH – H_2_O + H]^+^ at *m*/*z* 229 was owed to the existence of a glutathione group in the molecule. Hence, M18 was determined as the glutathione conjugation product of M26.

Metabolite M19 showed a [M + H]^+^ ion at *m*/*z* 343.12097 (C_16_H_23_O_6_S, 2.81 ppm) with 94 Da (CH_2_ + SO_3_) greater than M26. Among its ESI–MS^2^ spectrum, the monitoring of DPI [M – SO_3_ + H]^+^ at *m*/*z* 263 contributed to our speculation that M19 was a methylation and sulfonation product of M26.

The metabolite M43 eluted at 7.74 min possessed a [M + H]^+^ ion at *m*/*z* 306.17002 (C_17_H_24_O_4_N, 3.77 ppm), 57 Da (C_2_H_3_NO) larger than M26. Abundant product ions were observed at *m*/*z* 288, 260, and 242 in the ESI–MS^2^ spectrum, resulting from the continuous neutral loss of H_2_O versus CO. In addition, the DPI [M – Gly + H]^+^ at *m*/*z* 231 strongly demonstrated that M43 was the glycine conjugation product of M26. Comparable to the determination of M43, M47 (*m*/*z* 352.15772, C_18_H_26_O_4_NS), M50 (*m*/*z* 356.15262, C_17_H_26_O_5_NS), and M66 (*m*/*z* 394.16832, C_20_H_28_O_5_NS) were separately deduced to be cysteine conjugated, taurine conjugated and N-acetylcysteine conjugated esters. Correspondingly, M3 (*m*/*z* 324.18059, C_17_H_26_O_5_N) with 18 Da higher than M43 eluting at 4.07 min was qualified as the hydration product of M43.

Meanwhile, M11, M13, M14, M15, M24, and M29 with the same protonated [M + H]^+^ ions at *m*/*z* 247.13286 (C_15_H_19_O_3_, error ≤ ±3.00 ppm) were detected at 5.60, 5.65, 5.70, 5.84, 6.13, and 6.48 min, respectively. Since the fragment ions at *m*/*z* 229, 211, and 183 were generated by the sequential loss of H_2_O and CO, these six compounds, which were 16 Da higher than DL and shared the same cleavage behaviors, could be classified as the positional isomer products of DL hydroxylation. Furthermore, as 16 Da larger than M11 and manifested the same NLFs (*m*/*z* 263→*m*/*z* 245→*m*/*z* 227→*m*/*z* 199), M21 was recognized as a dihydroxylation product of DL.

Metabolites M48 and M49, which had the same [M + H]^+^ ion at *m*/*z* 408.14757 (C_20_H_26_O_6_NS, error ≤ ±3.70 ppm), were eluted at 8.50 and 8.55 min, respectively. Both were 161 Da (C_5_H_7_O_3_NS) higher than M11 with a high-intensity DPI [NAC – SH]^+^ at *m*/*z* 130 in the ESI–MS^2^ spectrum, so they were supposed to be N-acetylcysteine conjugation products of M11.

With the identical [M + H]^+^ ions at *m*/*z* 392.15266 (C_20_H_26_O_5_NS, error ≤ ±3.50 ppm) demonstrated, M36, M41, M44, M51, M62, and M65 were eluted at 7.16, 7.71, 7.82, 8.74, 9.71, and 10.12 min, respectively, which were 161 Da (C_5_H_7_O_3_NS) higher than that of DL. The DPIs at *m*/*z* 229[M – NAC + H]^+^, *m*/*z* 211[M – NAC – H_2_O + H]^+^, and *m*/*z* 201[M – NAC – CO + H]^+^ could conclusively indicate that they were N-acetylcysteine conjugation products of DL.

M22 and M30 were eluted at 5.98 and 6.51 min, respectively. They both gave the same protonated [M + H]^+^ ions at *m*/*z* 324.16522 (C_17_H_26_O_3_NS, error ≤ ±3.50 ppm), which was 68 Da (C_3_O_2_) lower than that of M36. As calculated by HRMS, those DPIs at *m*/*z* 159[C_12_H_15_]^+^, 157[C_12_H_13_]^+^, and 155[C_12_H_11_]^+^ were caused by the successive loss of NAC group and H_2_ molecules, whereby M22 and M30 were actually the products of M36 lost the A-ring (C_3_O_2_).

Metabolites M33, M38, and M42, which were individually obtained at elution times of 6.67, 7.19, and 7.74 min, produced [M + H]^+^ ions at *m*/*z* 229.12226 (C_15_H_17_O_2_, error ≤ ±3.00 ppm). The only 2 Da difference in mass (lower than DL) implied the intramolecular removal of one H_2_ molecule. In the MS/MS spectra, the NLFs of 18 Da (*m*/*z* 229→*m*/*z* 211→*m*/*z* 193) and 28 Da (*m*/*z* 211→*m*/*z* 183) resembled DL, illustrating them as the dehydrogenation products of DL. Likewise, M5, M7, M17, and M35, all of which gave rise to the same protonated [M + H]^+^ ion at *m*/*z* 227.10656 (C_15_H_15_O_2_, error ≤ ±3.50 ppm) with 4 Da less than DL, could be characterized as di-dehydrogenation products of DL.

M6, M8, M10, M34, and M56 all presented the same [M + H]^+^ ion at *m*/*z* 245.11716 (C_15_H_17_O_3_, error ≤ ±3.50 ppm), which was 16 Da higher than M33, hinting they were probably hydroxylation products of M33. Specific DPIs such as *m*/*z* 227, 209, and 199 observed in the ESI–MS^2^ spectrum were powerful corroboration for our reasoning. Otherwise, M39 (C_15_H_17_O_5_, 2.89 ppm), 48 Da larger than M33, was confirmed as a tri-hydroxylation product of M33 as well.

M23 yielded a [M + H]^+^ ion at *m*/*z* 390.13692 (C_20_H_24_O_5_NS, 3.03 ppm), which was 161 Da higher than that of M33. The observation of DPI[M – NAC + H]^+^ at *m*/*z* 227 undoubtedly pointed to M23 as the N-acetylcysteine conjugation product of M33.

In the positive ion mode, both M16 and M63 generated identical [M + H]^+^ ions at *m*/*z* 265.14336 (C_15_H_21_O_4_, error ≤ ±4.00 ppm), with retention times of 5.86 and 9.93 min, respectively, which was 34 Da (H_2_O_2_) higher than that of DL. As their ring double bond (RDB) values were 1 less than DL (6.5→5.5) as well as the DPIs at *m*/*z* 247, 229, and 201 all prompted us to conclude that an olefin double bond in DL was oxidized to dihydrodiol.

In the ESI–MS^1^ spectrum, the three isomers M4, M25, and M46 provided their protonated molecular ions at *m*/*z* 426.15802 (C_20_H_28_O_7_NS, error ≤ ±3.50 ppm), with retention times of 4.94, 6.31 and 8.19 min, respectively. Their *m*/*z* values were 161 Da higher than that of M16, where characteristic ions such as *m*/*z* 263, 245, 229, and 130 due to the presence of the NAC group were also visible in the MS^2^ spectrum, thus these three metabolites were N-acetylcysteine conjugation products of M16.

M20, 42 Da (C_3_H_6_) lower than M16, showed a [M + H]^+^ ion at *m*/*z* 223.0966 (C_12_H_15_O_4_, 3.52 ppm), with an elution time of 5.93 min. Generalizing the DPIs exhibited in its MS^2^ spectrum (*m*/*z* 205, 177) with NLFs (28, 18, and 14 Da), which were similar to DL, so it was assumed to be the product of M16 lost 3 molecules of methylene (CH_2_).

Metabolites M54, M58, M60, M67, and M70 with the same protonated molecular ion at *m*/*z* 213.12746 (C_15_H_17_O, error ≤ ±3.50 ppm) were separately eluted at 9.22, 9.58, 9.70, 10.38, and 13.38 min, 18 Da (H_2_O) lower than DL, demonstrating that the five compounds were dehydration products of DL. The *m*/*z* 185[C_14_H_17_]^+^ generated by the neutral loss of CO as observed in the MS^2^ spectrum implicitly suggested that one O atom (number 1) was lost in the DL structure, signifying that they were isomers with different intramolecular double bond positions. Accordingly, both M55 and M59 with identical protonated molecular ions at *m*/*z* 195.11689 (C_15_H_15_, error ≤ ±3.10 ppm), which were another 18 Da (H_2_O) lower than M54, were smoothly authenticated as the bi-dehydration products of DL.

M40 and M64 showed retention times of 7.70 and 10.09 min, respectively, and yielded the same theoretical [M + H]^+^ ions at *m*/*z* 185.13249 (C_14_H_17_, error ≤ ±3.50 ppm) with 28 Da (CO) lower than that of M54. The distinctive fragment ions at *m*/*z* 157, 143, and 129 resulted from the sequential loss of CH_2_ revealed that these two metabolites were the metabolic products of M54 by losing CO. Comparably, metabolite M68 (*m*/*z* 187.14816, C_14_H_19_) was 44 Da (CO_2_) lower than DL, which was clearly evidenced by the NLFs of 14 Da (*m*/*z* 187→*m*/*z* 159→*m*/*z* 145→*m*/*z* 131) in the ESI–MS^2^ spectrum as a product of DL lost CO+O.

### 2.5. Summary and Generalization of All Metabolites

**Figure 5:**
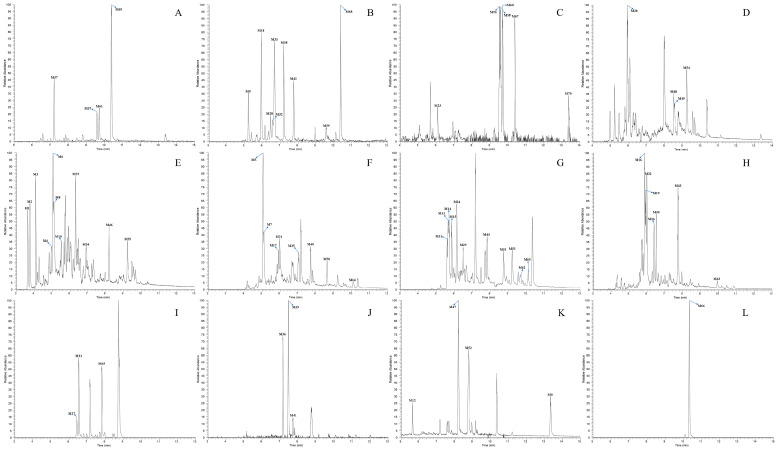
High-resolution extracted ion chromatograms of all DL metabolites in rat plasma, urine, and feces ((**A**–**C**) for plasma, (**D**–**I**) for urine and (**J**–**L**) for feces): (**A**) *m*/*z* 231.13796, 410.16322; (**B**) *m*/*z* 187.14816, 229.12226, 386.16319, 428.17379, 554.21676; (**C**) 213.12746, 245.11716, 390.13692; (**D**) 213.12746, 223.09662, 408.14757; (**E**) 195.11689, 245.11716, 324.18059, 368.15262, 426.15802; (**F**) 185.13249, 227.10656, 263.12776, 356.15262; (**G**) 231.13796, 247.13286, 392.15266; (**H**) 249.14856, 265.14336, 306.17002, 324.16522, 343.12097; (**I**) 410.16322; (**J**) 277.10697, 392.15266; (**K**) 231.13796, 352.15772, 368.15262, 410.16322; (**L**) 394.16832.

**Figure 6:**
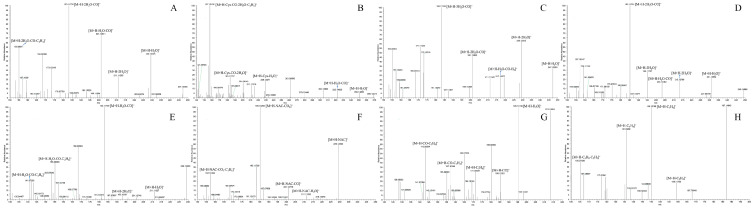
The ESI–MS^2^ spectra of eight typical metabolites: (**A**) M11; (**B**) M12; (**C**) M16; (**D**) M26; (**E**) M33; (**F**) M36; (**G**) M54; (**H**) M68.

Facilitated by the optimized strategy, a total of 71 DL metabolites including the parent drug (DL) were recognized and identified in the biological samples from rats. Among these metabolic products, 48 metabolites were attributed to plasma, 68 metabolites were discovered in urine, and just 24 metabolites were contained in feces. The chromatographic and mass spectrometric information from all the metabolites was summarized in Table 2, whereas the corresponding HREICs were visualized in Figure 5. Furthermore, the ESI–MS^2^ spectra of several representative metabolites were presented jointly in Figure 6.

### 2.6. Mapping of the Metabolic Network for DL in Rats

**Figure 7:**
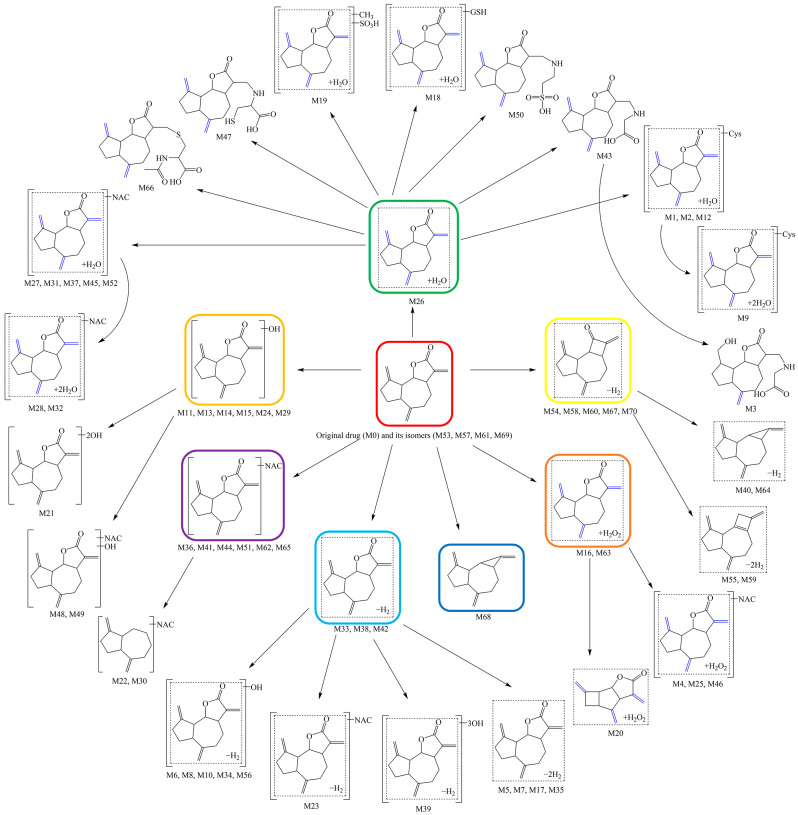
Mapping of the metabolic network for DL in rats.

Putting together all the metabolites detected and characterized, as well as analyzing their corresponding biotransformation reactions in rats, it was noted that the metabolic process of DL was hierarchical and progressive. At first, the parent drug (DL) was metabolized in rats to produce a few “core metabolites”, which were then continuously “branched out” in a “bifurcate-and-diverge” manner, until finally “flourishing” to integrate the special “stepwise radiation” metabolic network of DL. The network diagram was visualized in Figure 7.

Most certainly, the “core metabolites” including M11, M16, M26, M33, M36, M54, and M68, which were produced through hydroxylation, hydration, dehydration, dihydrodiolation, deoxidization, dehydrogenation, and N-acetylcysteine conjugation reactions, were crucial and might even be the necessary aspects for DL exerting its therapeutic effect, which was also in line with the drug metabolism clusters (DMCs) described in the available publications [23,24,25].

## 3. Discussion

In this study, a refined and optimal strategy was developed by integrating SPE technology, UHPLC–Q-Orbitrap HRMS, and various data-mining techniques, which was successfully applied to the metabolic study of an active ingredient of TCM—dehydrocostus lactone (DL) in rats. Compared with the previous reports [22], our results have greatly enlarged the metabolite database of DL and successfully mapped the corresponding metabolic network in vivo using the available analytical means, which will undoubtedly contribute to the current research regarding mechanisms and drug safety monitoring of DL.

As is well known, the most challenging problem in drug metabolism study is trace metabolite signal loss as a result of matrix effects and interference of endogenous substances in the analytical sessions. This unfavorable barrier may lead to the possibility that certain small-molecule metabolites with low-level but excellent pharmacological activities are unfortunately neglected. For example, acetaminophen (paracetamol), derived from the rapid de-ethylation of phenacetin in the liver, has an amazing antipyretic-analgesic effect [26]. As such, the suppression of background noise, wide-range and efficient data acquisition, and improvement of data processing techniques are always the directions of our endeavors.

Solid-phase extraction is a sample pre-treatment technology that is being developed quickly during recent years, which combines liquid-solid extraction (LSE) and column-liquid chromatography (CLC) techniques for sample separation, concentration, and purification [27]. When the samples pass through the stationary phase, the impurities and endogenous interferents can be effectively removed by adsorption, wash-out, and elution procedures, which will ultimately result in a purified collection containing the desirable target compounds. In contrast to the traditional protein precipitation and liquid–liquid extraction (LLE) approaches, SPE can be regarded as a “micro-scale chromatography”, which can minimize the matrix effects and ionization interferences with the integration of LC–MS/MS to achieve a “pseudo-two-dimensional chromatography”, particularly suitable for the selective enrichment of low-level small-molecule metabolites [28]. Additionally, the simplicity and rapidity of its operation make SPE destined to become a preferred tool for the optimization of data pre-acquisition strategies in drug metabolism analysis.

For UHPLC–MS/MS analysis, the conventional data-dependent acquisition mode has been broadly utilized in the majority of food and drug analysis studies because of its large-scale database acquisition capability with full scan followed by intensity-biased secondary (MS/MS) triggered successive acquisition patterns [29,30,31]. Nevertheless, the structural information obtained in this mode is redundant and contains a considerable number of unnecessary ion pairs, as well as a limited amount of MS^2^ information per data point, which may result in the absence of essential information and recognition difficulties for some compounds. Herein, based on the superior properties of Q-Orbitrap HRMS, the PIL-DE acquisition tactics coupled with FS-ddMS^2^ enabled either targeted monitoring of those ions under our interest and provoked MS^2^ collection (as literature findings and biotransformation reactions deduced), or dynamic exclusion on top of that to activate fragmentation for more low-abundance parent ions as well as to extend the coverage of sample constituents screening. As reported, PIL-DE could actually acquire more MS/MS information of trace ingredients [32], with the integration of FS-ddMS^2^ effectively realizing the high sensitivity and selectivity of HRMS for target constituents data acquisition while applying to drug metabolism analysis.

After obtaining the datasets by instrumental analysis, the mining of useful data from the wide-volume database up to high-efficiency screening and precise characterization of trace contents is a highly challenging topic. Previous studies mostly focused on the application of one or two methodologies, so that the screening and designation of trace metabolites were not accurate and complete enough. Mass defect filtering technology is designed to rapidly screen metabolites by imposing predetermined criteria around the mass defect of certain chosen template compounds (e.g., parent drug). However, with our practice, it was still difficult to cover numerous components in complex mixtures from just a single template, so a screening process that combined multiple templates (original drug, substructures, conjugates) was necessary. For this reason, a means of post-acquisition data handling merging MMDFs and HREICs was proposed, which allowed to conveniently exclude the majority of disturbing ions in the defined window from complicated matrices with further accurately pinpointing relevant metabolites at the ESI–MS^1^ level. Moreover, compounds with the same parent nucleus and similar backbones display analogous fragmentation behaviors under high-energy collisional dissociation (HCD), thus providing the common DPIs and NLFs for structural elaboration, which could further serve as potent auxiliary tools for metabolites identification.

In general, the above-mentioned refined strategy was mainly aimed at the signals overwhelmed, screening omission, and structures mis-definition of trace metabolites caused by severe matrix effects, narrow acquisition coverage, and massive data processing difficulties in drug metabolism LC–MS/MS analysis. In fact, in the present study, the entire three phases of the drug metabolism experiment—“pre-acquisition, in-acquisition, and post-acquisition”—were improved, resulting in an advanced strategy that facilitated the high-efficiency and high-velocity collection for the targeted recognition of major-to-trace metabolites as well as the precise and comprehensive screening to enrich the small-molecule metabolites library. Following our verification, the strategy was promising to be well suited for in vivo metabolic process profiling studies of any Chinese medicine prescriptions or monomeric compounds.

## 4. Materials and Methods

### 4.1. Reagents and Chemicals

The dehydrocostus lactone (DL) reference standard was purchased from Chengdu Must Bio-technology Co., Ltd. (Sichuan, China). Its structure was well demonstrated by spectral matching with published literature (ESI–MS, ^1^H, and ^13^C NMR), of which the purity was no less than 98% according to HPLC-UV analysis. MS grade acetonitrile and methanol were obtained from Thermo Fisher Scientific (Fair Lawn, NJ, USA), while HPLC grade formic acid (FA) was purchased from Merck KGaA (Darmstadt, Germany). All additional chemicals of analytical grade were accessible at the work station, Beijing Chemical Works (Beijing, China). The deionized water (ddH_2_O) used throughout the analytical workflow was prepared in purification with the Milli-Q Synthesis System (Millipore, Billerica, MA, USA). Sep-Pak^®^Vac C18 solid-phase extraction cartridges utilized for the pre-treatment of all biological samples were obtained from Waters (Milford, MA, USA).

### 4.2. Animals and Drug Administration

Six Sprague Dawley (SD) rats were acquired from SPF Biotechnology Co. Ltd. (Beijing, China). All animals were living under stable controlled conditions in the barrier environment (temperature: 23 ± 2 °C; relative humidity: 55±10%; alternating day/night time: 12/12 h) with free access to food and water. Following one week of continuous acclimatization to the environment, the rats were randomly divided into two groups: the control group (*n* = 3) and the drug-treated group (*n* = 3). In this case, the drug-treated group was given DL, which was suspended in 0.5% sodium carboxymethyl cellulose (CMC-Na) by gavage at a dose of 300 mg/kg, while the control group was orally administered an equal amount of 0.5% CMC-Na solution. The overall animal experimental program and details were authorized by the Institutional Animal Care and Use Committee at the Beijing University of Chinese Medicine, as well as all procedures were conducted in accordance with the Guide for the Care and Use of Laboratory Animals (USA National Research Council, 1996).

### 4.3. Biological Samples Collection

All the rats were fasted for 12 h but were allowed to drink water freely before drug administration. After gastric administration, approximately 0.8 mL of blood was gathered from the orbital venous plexus in each rat at 0.5, 1, 2, 4, and 6 h followed by transferring to heparin sodium anticoagulation EP tubes, which were then centrifuged for 15 min (3500 rpm, 4 °C) to separate plasma.

Further, all urine and feces excreted by every individual rat within 24 h post-dosing were also collected. Urine was likewise centrifuged for 15 min (3500 rpm, 4 °C) to accumulate supernatant, whereas the feces were dried, ground, and crushed, of which 0.5 g was taken from each group and plunged into 70% ethanol (*m/v* = 1:10), and the supernatant was extracted by ultrasonication for 30 min, followed by 15 min centrifugation (3500 rpm, 4 °C).

Eventually, homogeneous biological samples (i.e., plasma, urine, and feces) from the same group were co-mingled into an aggregate set and stored at −80 °C.

### 4.4. Samples Pre-Treatment—The SPE Method

Pending instrumental analysis, SPE technology was adopted for rapid separation, purification, and concentration of all biological samples. At first, the SPE cartridges were sequentially flushed with 5 mL of ddH_2_O and 5 mL of methanol for activation and equilibration. Next, 1 mL of biological samples (plasma, urine, and feces) were separately loaded into the cartridges allowing natural adsorption. After that, the cartridges were washed again with 5 mL of ddH_2_O to remove most of the matrix interferents. Last, the cartridges were eluted twice using 1 mL of methanol so that a total of 2 mL eluate (target analytes contained) was collected, which was further evaporated by blow-drying with nitrogen under room temperature. The final residues were re-solubilized in 2% acetonitrile solution (100 μL) with centrifugation for 15 min (12,000 rpm, 4 °C) after which the supernatants were collected for UHPLC–HRMS analysis.

### 4.5. Instrument and Conditions

All LC–MS/MS analytical procedures were accomplished on a UHPLC–Q-Orbitrap high-resolution mass spectrometer (Thermo Scientific, Bremen, Germany) equipped with an ESI source. The chromatographic separation was carried out using a Waters ACQUITY UPLC HSS T3 column (2.1 mm × 100 mm i.d., 1.8 μm; Waters Corporation, Milford, MA, USA) through a mobile phase system of 0.1% FA aqueous solution (A)-acetonitrile (B), with a flow rate of 0.30 mL/min at 35 °C. The specific gradient elution program was adjusted as follows: 0–1 min, 2% B; 1–3 min, 2–20% B; 3–10 min, 20–60% B; 10–17 min, 60–80% B; 17–19 min, 80–100% B; 19–21 min, 100% B; 21–22 min, 100–2% B; 22–25 min, 2% B. The injection volume was 2 μL.

The operational parameters for Q-Orbitrap HRMS in the positive ion mode were optimized as follows: spray voltage, 3500 V; sheath gas flow rate, 50 arb; auxiliary gas flow rate, 10 arb; ion transfer tube temperature, 350 °C. All objective compositions were detected and analyzed with a resolution of 60,000 in the scan range of *m*/*z* 100–1000. Collision Energy Type was chosen as “Normalized” with HCD collision energies (CE) being set to 30%, 50%, and 70%. The dynamic exclusion (DE) assignments were configured as follows: the repeat count was 5, the dynamic repeat time was 30 s, and the dynamic exclusion duration was 60 s.

### 4.6. Data Processing

All LC–MS/MS data were extracted and processed on an Xcalibur 4.3 workstation. For the purpose of securing as much information as possible concerning ions fragmentation of small-molecule metabolites, we selected the peaks with an intensity threshold exceeding 50,000 for screening and identification. In the prediction interface for the formulated molecular formula, the parameters of “elements in use” were set up as: C [0–30], H [0–60], O [0–15], S [0–5], N [0–5] and ring double bond (RDB) equivalent value [0–15]. On the basis of accurate mass measurements and predicted elemental composition, the mass error between theoretical and experimental values of metabolites was fixed to within ±5 ppm.

In addition, MetWorks (version 1.3) software (Thermo Scientific, Waltham, MA, USA) was employed to implement the MMDF function along with the NLF capability for extensive screening of metabolites, which combined with Mass Frontier (version 8.0) software (Thermo Scientific, Waltham, MA, USA) to predict the fragmentation behaviors of metabolites and attribute fragment ions, ultimately allowing for precise structural characterization of metabolites.

## 5. Conclusions

In the current study, an advanced strategy integrating solid-phase extraction technology, UHPLC–Q-Orbitrap HRMS analysis, and multidimensional data-mining techniques was developed, which successfully achieved rapid and in-depth targeted recognition and accurate identification of 71 metabolites from DL. Furthermore, based on the step-by-step profiling of biotransformation reactions (i.e., hydration, hydroxylation, dehydrogenation, dehydration, N-acetylcysteine conjugation, etc.), the “stepwise radiation” metabolic network of DL in rats was resoundingly mapped with the discovery of drug metabolic cluster centers such as M11, M16, M26, M33, M36, M54, and M68, which will hopefully accelerate the mechanism exploration and exploitation of DL in the medical community. Most importantly, to the best of our knowledge, this study was the first report on the comprehensive metabolism study of DL in a bio-organism, and the proposed refined strategy is prospectively and widely applicable to the metabolic profile elucidation of active ingredients from TCM in vivo.

## Figures and Tables

**Table 1:** The principal screening templates in the MMDF method.

Number	Template Definition	Molecular Formula	*m*/*z* Value
1	Original drug (DL)	C_15_H_18_O_2_	230.13013
	N-acetylcysteine conjugation	C_20_H_25_O_5_NS	391.14480
	Sulfate conjugation	C_15_H_18_O_5_S	310.08695
2	Hydrated DL	C_15_H_20_O_3_	248.14070
	N-acetylcysteine conjugation	C_20_H_27_O_6_NS	409.15536
	Sulfate conjugation	C_15_H_20_O_6_S	328.09751
3	Dehydrated DL	C_15_H_16_O	212.11957
	N-acetylcysteine conjugation	C_20_H_23_O_4_NS	373.13423
	Sulfate conjugation	C_15_H_16_O_4_S	292.07638

**Table 2:** Summary of DL metabolites in rat plasma, urine, and feces.

Peak	t_R_/Min	Formula [M+H]^+^	Theoretical Mass *m*/*z*	Experimental Mass *m*/*z*	Error (ppm)	MS/MS Fragment Ions	Identification/Reactions	P	U	F
M0	13.34	C_15_H_19_O_2_	231.13796	231.13864	2.96	MS^2^[231]:185(100),143(51),157(25),213(16),195(13)	Dehydrocostus lactone	+	+	+
M1	3.63	C_18_H_26_O_5_NS	368.15262	368.15439	4.81	MS^2^[368]:157(100),183(22),229(21),322(7),350(6)	Hydration, cysteine-S-conjugation	—	+	—
M2	3.75	C_18_H_26_O_5_NS	368.15262	368.15445	4.97	MS^2^[368]:157(100),183(26),229(19),322(7),350(5)	Hydration, cysteine-S-conjugation	—	+	—
M3	4.07	C_17_H_26_O_5_N	324.18059	324.18152	2.99	MS^2^[324]:324(100),306(9),288(4),270(1),231(1),249(1)	Bi-hydration, glycine conjugation to ester	—	+	—
M4	4.94	C_20_H_28_O_7_NS	426.15802	426.15948	3.24	MS^2^[426]:130(100),199(33),227(25),245(12),217(11)	Dihydrodiolation, N-acetylcysteine-S-conjugation	+	+	—
M5	5.04	C_15_H_15_O_2_	227.10656	227.10732	2.92	MS^2^[227]:181(100),153(15),167(7),209(4),199(3)	Di-dehydrogenation	+	+	—
M6	5.04	C_15_H_17_O_3_	245.11716	245.11794	2.93	MS^2^[245]:181(100),199(31),155(19),227(14),209(6)	Dehydrogenation, hydroxylation	+	+	—
M7	5.11	C_15_H_15_O_2_	227.10656	227.10735	3.06	MS^2^[227]:181(100),153(14),199(11),167(6),209(4)	Di-dehydrogenation	+	+	—
M8	5.11	C_15_H_17_O_3_	245.11716	245.11792	2.85	MS^2^[245]:181(100),199(36),227(23),155(22),209(6)	Dehydrogenation, hydroxylation	+	+	—
M9	5.20	C_18_H_28_O_6_NS	386.16319	386.16440	3.15	MS^2^[386]:157(100),229(74),368(43),201(35),247(7)	Bi-hydration, cysteine-S-conjugation	+	+	—
M10	5.51	C_15_H_17_O_3_	245.11716	245.11792	3.18	MS^2^[245]:181(100),199(47),227(20),155(18),209(15)	Dehydrogenation, hydroxylation	+	+	+
M11	5.60	C_15_H_19_O_3_	247.13286	247.13354	2.71	MS^2^[247]:183(100),201(61),155(56),229(48),211(26)	Hydroxylation	+	+	—
M12	5.63	C_18_H_26_O_5_NS	368.15262	368.15344	2.23	MS^2^[368]:157(100),183(22),229(18),322(8),350(5)	Hydration, cysteine-S-conjugation	+	—	+
M13	5.65	C_15_H_19_O_3_	247.13286	247.13353	2.67	MS^2^[247]:183(100),201(66),155(57),229(48),211(25)	Hydroxylation	+	+	—
M14	5.70	C_15_H_19_O_3_	247.13286	247.13348	2.46	MS^2^[247]:183(100),201(56),229(55),155(52),211(21)	Hydroxylation	+	+	—
M15	5.84	C_15_H_19_O_3_	247.13286	247.13351	2.59	MS^2^[247]:201(100),229(91),183(57),155(53),211(11)	Hydroxylation	+	+	—
M16	5.86	C_15_H_21_O_4_	265.14336	265.14413	2.62	MS^2^[265]:183(100),229(57),201(37),247(31),217(16)	Dihydrodiolation	+	+	—
M17	5.91	C_15_H_15_O_2_	227.10656	227.10741	3.32	MS^2^[227]:181(100),153(23),199(20),209(6),167(5)	Di-dehydrogenation	—	+	—
M18	5.92	C_25_H_36_O_9_N_3_S	554.21676	554.21790	2.21	MS^2^[554]:157(100),229(80),185(52),211(20),201(9)	Hydration, glutathione conjugation	+	—	—
M19	5.92	C_16_H_23_O_6_S	343.12097	343.12195	2.81	MS^2^[343]:173(100),263(80),227(71),199(57),245(26)	Hydration, methylation, sulfonation	—	+	—
M20	5.93	C_12_H_15_O_4_	223.09662	223.09727	3.52	MS^2^[223]:121(100),135(80),181(54),177(12),205(6)	Dihydrodiolation, loss of 3CH_2_	+	+	+
M21	5.98	C_15_H_19_O_4_	263.12776	263.12860	3.10	MS^2^[263]:199(100),227(30),181(27),245(26),217(12)	Dihydroxylation	—	+	—
M22	5.98	C_17_H_26_O_3_NS	324.16522	324.16385	3.27	MS^2^[324]:157(100),155(24),159(10),129(9),143(3)	Loss of C_3_O_2_, N-acetylcysteine-S-conjugation	—	+	+
M23	6.08	C_20_H_24_O_5_NS	390.13692	390.13815	3.03	MS^2^[390]:183(100),181(62),227(31),199(3),209(1)	Dehydrogenation, N-acetylcysteine-S-conjugation	+	+	—
M24	6.13	C_15_H_19_O_3_	247.13286	247.13354	2.71	MS^2^[247]:183(100),201(64),229(43),155(38),211(17)	Hydroxylation	+	+	—
M25	6.31	C_20_H_28_O_7_NS	426.15802	426.15942	3.10	MS^2^[426]:130(100),183(49),229(47),157(32),211(7)	Dihydrodiolation, N-acetylcysteine-S-conjugation	—	+	—
M26	6.38	C_15_H_21_O_3_	249.14856	249.14925	2.93	MS^2^[249]:185(100),195(28),231(21),213(18),203(16)	Hydration	+	+	—
M27	6.44	C_20_H_28_O_6_NS	410.16322	410.16449	3.18	MS^2^[410]:130(100),229(62),201(37),185(22),247(2)	Hydration, N-acetylcysteine-S-conjugation	+	+	—
M28	6.45	C_20_H_30_O_7_NS	428.17379	428.17484	2.55	MS^2^[428]:130(100),229(47),201(31),247(4),386(1)	Bi-hydration, N-acetylcysteine-S-conjugation	+	+	+
M29	6.48	C_15_H_19_O_3_	247.13286	247.13361	2.99	MS^2^[247]:183(100),201(91),229(62),155(50),211(31)	Hydroxylation	+	+	—
M30	6.51	C_17_H_26_O_3_NS	324.16522	324.16388	3.36	MS^2^[324]:157(100),155(19),129(17),143(8),159(7)	Loss of C_3_O_2_, N-acetylcysteine-S-conjugation	—	+	—
M31	6.54	C_20_H_28_O_6_NS	410.16322	410.16428	2.67	MS^2^[410]:130(100),229(44),201(31),185(18),247(3)	Hydration, N-acetylcysteine-S-conjugation	+	+	—
M32	6.56	C_20_H_30_O_7_NS	428.17379	428.17459	1.96	MS^2^[428]:130(100),229(75),386(34),201(30),247(4)	Bi-hydration, N-acetylcysteine-S-conjugation	+	+	+
M33	6.67	C_15_H_17_O_2_	229.12226	229.12296	2.85	MS^2^[229]:183(100),155(39),141(19),211(8),193(4)	Dehydrogenation	+	+	+
M34	6.90	C_15_H_17_O_3_	245.11716	245.11794	2.93	MS^2^[245]:181(100),217(67),199(12),227(5),155(4)	Dehydrogenation, hydroxylation	+	+	—
M35	7.04	C_15_H_15_O_2_	227.10656	227.10742	3.36	MS^2^[227]:181(100),153(14),167(6),209(5),199(2)	Di-dehydrogenation	+	+	—
M36	7.16	C_20_H_26_O_5_NS	392.15266	392.15356	2.40	MS^2^[392]:185(100),229(60),157(26),201(12),211(5)	N-acetylcysteine-S-conjugation	+	+	+
M37	7.19	C_20_H_28_O_6_NS	410.16322	410.16412	2.28	MS^2^[410]:229(100),185(89),157(54),130(35),201(30)	Hydration, N-acetylcysteine-S-conjugation	+	+	+
M38	7.19	C_15_H_17_O_2_	229.12226	229.12288	2.50	MS^2^[229]:183(100),155(25),141(13),211(7),193(5)	Dehydrogenation	+	+	+
M39	7.46	C_15_H_17_O_5_	277.10697	277.10785	2.89	MS^2^[277]:121(100),217(16),189(13),231(9),259(3)	Dehydrogenation, tri-hydroxylation	—	—	+
M40	7.70	C_14_H_17_	185.13249	185.13304	3.04	MS^2^[185]:185(100),143(87),157(51),129(49),131(6)	Dehydration, loss of CO	+	+	—
M41	7.71	C_20_H_26_O_5_NS	392.15266	392.15381	3.03	MS^2^[392]:185(100),229(44),157(14),201(2),211(1)	N-acetylcysteine-S-conjugation	+	+	+
M42	7.74	C_15_H_17_O_2_	229.12226	229.12279	2.11	MS^2^[229]:183(100),155(33),129(13),141(10),211(7)	Dehydrogenation	+	+	+
M43	7.74	C_17_H_24_O_4_N	306.17002	306.17114	3.77	MS^2^[306]:159(100),260(18),242(4),231(2),288(1)	Hydration, glycine conjugation to ester	+	+	—
M44	7.82	C_20_H_26_O_5_NS	392.15266	392.15384	3.11	MS^2^[392]:185(100),229(78),157(38),201(18),211(7)	N-acetylcysteine-S-conjugation	+	+	+
M45	7.82	C_20_H_28_O_6_NS	410.16322	410.16446	3.11	MS^2^[410]:229(100),185(79),157(45),130(30),201(23)	Hydration, N-acetylcysteine-S-conjugation	+	+	—
M46	8.19	C_20_H_28_O_7_NS	426.15802	426.15955	3.40	MS^2^[426]:145(100),130(10),217(8),245(3),263(1)	Dihydrodiolation, N-acetylcysteine-S-conjugation	—	+	—
M47	8.20	C_18_H_26_O_4_NS	352.15772	352.15842	2.03	MS^2^[352]:159(100),213(31),185(13),306(10),231(5)	Hydration, cysteine conjugation to ester	+	+	+
M48	8.50	C_20_H_26_O_6_NS	408.14757	408.14896	3.49	MS^2^[408]:130(100),157(32),229(26),183(24),199(7)	Hydroxylation, N-acetylcysteine-S-conjugation	—	+	—
M49	8.55	C_20_H_26_O_6_NS	408.14757	408.14902	3.64	MS^2^[408]:130(100),157(33),183(22),229(17),211(7)	Hydroxylation, N-acetylcysteine-S-conjugation	—	+	—
M50	8.62	C_17_H_26_O_5_NS	356.15262	356.15369	3.00	MS^2^[356]:229(100),201(35),187(21),183(5),217(3)	Hydration, taurine conjugation to ester	—	+	—
M51	8.74	C_20_H_26_O_5_NS	392.15266	392.15378	2.96	MS^2^[392]:185(100),157(33),229(10),211(6),201(2)	N-acetylcysteine-S-conjugation	—	+	+
M52	8.77	C_20_H_28_O_6_NS	410.16322	410.16409	2.21	MS^2^[410]:130(100),159(36),185(12),213(8),231(2)	Hydration, N-acetylcysteine-S-conjugation	—	+	+
M53	9.22	C_15_H_19_O_2_	231.13796	231.13858	2.70	MS^2^[231]:185(100),143(52),157(29),213(17),195(16)	Dehydrocostus lactone isomer	—	+	—
M54	9.22	C_15_H_17_O	213.12746	213.12802	2.95	MS^2^[213]:195(100),143(61),171(32),157(30),185(29)	Dehydration	—	+	—
M55	9.22	C_15_H_15_	195.11689	195.11739	2.89	MS^2^[195]:195(100),165(66),167(12),155(5),193(3)	Bi-dehydration	—	+	—
M56	9.51	C_15_H_17_O_3_	245.11716	245.11804	3.34	MS^2^[245]:181(100),155(5),227(4),199(3),209(1)	Dehydrogenation, hydroxylation	+	+	+
M57	9.58	C_15_H_19_O_2_	231.13796	231.13857	2.66	MS^2^[231]:185(100),143(50),157(29),213(16),195(15)	Dehydrocostus lactone isomer	+	+	—
M58	9.58	C_15_H_17_O	213.12746	213.12805	3.09	MS^2^[213]:195(100),143(55),157(30),171(28),185(20)	Dehydration	+	+	—
M59	9.58	C_15_H_15_	195.11689	195.11742	3.04	MS^2^[195]:195(100),165(80),167(11),155(7),193(4)	Bi-dehydration	+	+	—
M60	9.70	C_15_H_17_O	213.12746	213.12810	3.32	MS^2^[213]:195(100),143(54),157(28),171(27),185(20)	Dehydration	+	+	—
M61	9.70	C_15_H_19_O_2_	231.13796	231.13866	3.04	MS^2^[231]:185(100),143(49),157(26),213(15),195(14)	Dehydrocostus lactone isomer	+	+	—
M62	9.71	C_20_H_26_O_5_NS	392.15266	392.15390	3.26	MS^2^[392]:229(100),157(24),201(24),211(15),185(12)	N-acetylcysteine-S-conjugation	—	+	—
M63	9.93	C_15_H_21_O_4_	265.14336	265.14444	3.79	MS^2^[265]:145(100),229(81),201(72),183(56),247(16)	Dihydrodiolation	—	+	—
M64	10.09	C_14_H_17_	185.13249	185.13310	3.37	MS^2^[185]:185(100),143(79),157(54),129(42),131(6)	Dehydration, loss of CO	—	+	—
M65	10.12	C_20_H_26_O_5_NS	392.15266	392.15393	3.34	MS^2^[392]:157(100),185(61),229(26),143(16),211(15)	N-acetylcysteine-S-conjugation	—	+	—
M66	10.34	C_20_H_28_O_5_NS	394.16832	394.16916	2.26	MS^2^[394]:159(100),185(81),213(33),231(27),352(5)	Hydration, N-acetylcysteine conjugation to ester	+	+	+
M67	10.38	C_15_H_17_O	213.12746	213.12798	2.76	MS^2^[213]:195(100),143(48),157(32),171(24),185(17)	Dehydration	+	+	+
M68	10.38	C_14_H_19_	187.14816	187.14859	2.47	MS^2^[187]:187(100),145(99),131(75),105(40),159(18)	Loss of CO+O	+	+	+
M69	10.38	C_15_H_19_O_2_	231.13796	231.13855	2.57	MS^2^[231]:185(100),143(61),157(33),129(28),213(18)	Dehydrocostus lactone isomer	+	+	+
M70	13.38	C_15_H_17_O	213.12746	213.12808	3.23	MS^2^[213]:195(100),143(46),157(27),171(23),185(17)	Dehydration	+	+	+

Note: t_R_: retention time; U: urine; P: plasma; F: feces; +: detected; —: undetected.

## Data Availability

The research data in this study are available from the corresponding author upon reasonable request.
